# How do patients with systemic autoimmune rheumatic disease perceive the use of their medications: a systematic review and thematic synthesis of qualitative research

**DOI:** 10.1186/s41927-018-0017-8

**Published:** 2018-04-02

**Authors:** Hans Haag, Tim Liang, J. Antonio Avina-Zubieta, Mary A. De Vera

**Affiliations:** 1 University of British Columbia, Faculty of Pharmaceutical Sciences, 2405 Wesbrook Mall, Vancouver, BC V6T 1Z3 Canada; 2 Arthritis Research Canada, Richmond, BC Canada; 3 Collaboration for Outcomes Research and Evaluation, Vancouver, BC Canada; 4 Department of Medicine, Division of Rheumatology, University of British Columbia, Faculty of Medicine, Vancouver, BC Canada

**Keywords:** Concordance, Medication use, Qualitative research, Systematic review, Systematic autoimmune rheumatic disease, Systemic lupus erythematosus

## Abstract

**Background:**

Pharmacotherapy is paramount to the management of systemic autoimmune rheumatic diseases (SARDs), yet there is sub-optimal adherence and limited adherence interventions. To understand how to better support patients’ medication use, our two-fold objectives were: 1) to conduct a systematic review of qualitative research studies of medication taking among SARD patients; and 2) to thematically synthesize qualitative research studies to obtain SARD patients’ perspectives and experiences with medication use.

**Methods:**

We conducted a search of MEDLINE, EMBASE, Cumulative Index to Nursing and Allied Health Literature, and Social Sciences Citation Index databases to identify qualitative research studies exploring views on medication use among patients with SARDs, their healthcare providers, or caregivers. We used thematic synthesis to combine data from selected studies, and identify analytical themes on SARD patients’ perspectives and experiences with medication use.

**Results:**

Our systematic review identified 18 studies. Thematic synthesis identified seven analytical themes: 1) effects of medications on emotional and social well-being, 2) impacts of healthcare provider relationships on treatment, 3) gaining control over treatment, 4) fear and concern with side effects of treatment, 5) understanding the importance of treatment, 6) practical barriers to taking medication, and 7) motivation towards adherence to treatment.

**Conclusion:**

This systematic review and thematic synthesis contributes to better understanding of SARDs patients’ perspectives on medication use. Given the paucity of existing adherence interventions targeting this patient population, our study has certain practical implications for care, namely the need to address emotional and social impacts of medication use and the necessity of establishing a meaningful and trusting professional relationship with patients.

## Background

Systemic autoimmune rheumatic diseases (SARDs) are a group of rare inflammatory conditions that are associated with autoimmune dysregulation leading to disability, organ failure and premature mortality [1]. SARDs include systemic lupus erythematosus (SLE), systemic sclerosis (SSc), inflammatory myositis (i.e. polymyositis and dermatomyositis), Sjogren’s syndrome, and systemic vasculitides (giant cell arteritis, eosinophilic granulomatosis with polyangiitis, granulomatosis with polyangitis, polyarteritis nodosa). As there is no cure for SARDs, long-term pharmacotherapy is paramount to their management. In the acute phase of treatment, glucocorticoids are considered first-line therapy, but their side effect profile limits their chronic use so antimalarials and immunosuppressive medications are essential to long-term SARD therapy [2, 3].

However, with chronic therapies like those used to manage SARDs, adherence is important [4]. A 2017 systematic review of 11 observational studies in SLE reported adherence rates ranging from 25 to 57% [4]. With respect to other SARDs, studies reported adherence rates of 42% to treatments overall [5] and 64.1% to glucocorticoids [6], respectively among patients with SSc. Yet despite the evidence for suboptimal adherence in SARDs, there remains a scarcity of feasible adherence interventions - a 2015 systematic review identified only three interventions, all aimed at SLE, which showed inconsistent effects [7–9].

Indeed, there is need for more research on interventions targeting adherence across all SARDs, particularly incorporating patient-centered approaches to development and evaluation [10, 11]. Qualitative research provides a means to informing such interventions through gathering of patients’ perspectives, views, and opinions on medication use [12]. Synthesis of primary research studies addresses inherent limitations including small sample sizes and issues with generalizability [13]. In 2013, Sutanto et al. reported their thematic synthesis of qualitative studies, published between 1993 and 2012 on patients’ experiences of living with SLE, which identified five themes including that of *treatment adherence* [14]. However, adherence itself is encompassed by “medication taking”, a broader term that encompasses patients’ behaviours, attitudes and knowledge of medications [15]. There remains to date no qualitative synthesis specifically focusing on experiences with medication use among SARD patients. To update prior work on SLE with a focus on medication taking as well as gather information on lesser-studied SARDs, our two-fold objectives were to: 1) conduct an updated systematic review of qualitative research studies of medication taking among SARD patients; and 2) thematically synthesize qualitative research studies to obtain SARD patients’ perspectives and experiences with medication use.

## Methods

### Search strategy

We conducted a search of MEDLINE (1 Jan 1946 – 12 Jun 2017), EMBASE (1 Jan 1974 – 12 Jun 2017), Cumulative Index to Nursing and Allied Health Literature (1982 – 12 Jun 2017), and Social Sciences Citation Index (1965 – 12 Jun 2017) databases. Our search strategy employed mapped subject headings together with keywords (expressed as truncated wildcards where possible [16]) for unindexed terms relating to the concepts of SARDs, qualitative methods, and medication use (see Additional file 1). Inclusion criteria were: 1) study sample of patients with SARDs, their healthcare providers, or caregivers; 2) study describing these individuals’ views on medication taking specifically for disease management; 3) qualitative study design; 4) primary research article; and 5) English language of publication. As one of our objective was to update the prior systematic review of patient experiences with SLE [14] but with a focus on medication taking, we considered studies in SLE published 2013 and onwards, as identified in our search strategy, for the systematic review.

### Study selection and data extraction

Two authors (HH and TL) reviewed titles and abstracts for inclusion of published studies meeting systematic review criteria. Disagreement was resolved by consensus. Additional articles meeting our inclusion criteria that were not captured by the search strategy were obtained by a hand-search of relevant bibliographies. We extracted the following information from included studies: year of publication, country, disease studied, patient characteristics (i.e. type of SARDs, age, sex), and data collection and analysis methods.

### Thematic synthesis

For thematic synthesis, we included studies identified in our systematic review as well as studies from the aforementioned prior systematic review of patient experiences with SLE [14] that met our inclusion criteria. Findings of each article (including text, tables, and any available supplementary material) were imported verbatim into NVivo Version 11.4.1.1064 (64-bit). Thematic synthesis comprised three steps [17]. First, we coded each line of extracted data focusing on experiences and perspectives of SARD patients with taking their medications according to meaning and content. As subsequent articles were analyzed, we translated concepts across studies, developing new codes as necessary. We subsequently established a coding framework, which we applied to articles to ensure that all concepts were integrated. Second, we examined similarities and differences between the codes and organized them into a hierarchical structure to derive descriptive themes. Third, we mapped the interrelation between descriptive themes to generate theory-driven analytical themes - a cyclical process that involved generation of analytical themes independently by two authors, group discussion, re-examination and modification as necessary, and another group discussion of modified themes, until no new analytical themes emerged [18].

## Results

### Characteristics of studies included in the systematic review

**Fig. 1:**
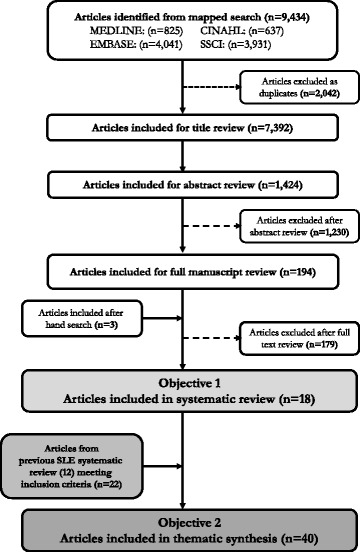
Study Flow for a) Systematic Review and b) Thematic Synthesis of SARD Patients’ Perspectives and Experiences with Medication Use

Our search strategy as shown in Fig. 1 identified 7392 articles after the removal of duplicates, with 197 forwarded for full-text review. Of note as one of our aims was to update the prior systematic review of patient experiences with SLE [14] but with a focus on medication use, we only included studies in SLE published 2013 and onwards. In the end, 18 studies met all inclusion criteria for our systematic review (Table 1). With respect to types of SARDs, eight studies were based on SLE only, three studies were based on other SARDs including SSc, Sjogren’s syndrome and a mixed sample involving eosinophilic granulomatosis with polyangiitis, granulomatosis with polyangitis, and polyarteritis nodosa, and seven studies were based on mixed samples including patients with SLE, other SARDs, and other inflammatory arthritides. Fourteen studies reported mean disease duration, which ranged from 2.3 to 20.4 years, and three studies reported mean age at diagnosis which ranged from 12.5 to 35 years old [19–34]. Two studies however did not report any information about how long their study participants had a SARD diagnosis [35, 36]. Finally, three studies included patients as well as healthcare providers and caregivers [21, 24, 31].

**Table 1:** Characteristics of Qualitative Studies Included in the Systematic Review

Author	Year	Country	Disease^a^ (N)	Mean Age	Sex (%F)	Study Methods	Aim
Studies with SLE only							
Feldman et al. [22]	2013	USA	**SLE** (29)	51 (range 25–81)	100	Focus groups	To determine interventions perceived to improve care
Mazzoni et al. [29]	2014	Italy	**SLE** (9)	50.6 (range 41–61)	100	Face-to-face interview	To explore the experiences of problematic support from SLE patients’ perspective
Neville et al. [31]	2014	Canada	**SLE** (29)	- (range 18–69)	93.1	Focus groups	To identify the informational and resource needs of persons with lupus
Singh et al. [32]	2015	USA	**SLE** (52)	40.6 (range not given)	–	Nominal group technique	To determine what factors facilitated decisional processes involving medications
Hale et al. [23]	2015	USA	**SLE** (15)	41.7 (range 22–57)	93.3	Semi-structured interviews	To explore the interactions between body image, self-image, medication use, and adherence to medication use in SLE
Brennan et al. [36]	2016	UK Ireland	**SLE** (133)	–	–	Open-ended questionnaire	To explore perceptions of the quality and impact of social support for individuals with SLE
Tunnicliffe et al. [34]	2016	Australia	**SLE** (26)	–	92	Focus groups Semi-structured interviews	To describe the experiences, perspectives, and health care needs of adolescents and young adults with SLE
Mathias et al. [27]	2017	USA	**SLE** (33)	47.0 (range 24–71)	97	Semi-structured interviews	To develop a PRO measure which can be used to assess general impact (baseline burden), benefits, side effects, and impacts associated with oral steroid use over time in patients with SLE.
Studies with other SARDs							
Mooney et al. [30]	2013	UK	CSS (3) GPA (10) PAN (2)	63.6 (range 39–80)	66.7	Focus groups Face-to-face interviews	To explore the informational needs of patients with AAV
Suarez-Almazor et al. [33]	2015	USA	**SSc** (19)	49 (range not given)	90	Focus groups Face-to-face interview	To identify subjective domains that need to be measured in order to improve patient-centered outcome measurement for SSc.
Lackner et al. [25]	2017	Austria	**Primary Sjogren’s syndrome** (20)	62 (range not given)	–	Focus groups	To explore the perspectives and aspects of HRQL in patients with PSS in a qualitative manner.
Studies with mixed patient populations including SLE and other SARDs							
Alparslan et al. [35]	2010	Turkey	Arthritis (60) Renal diseases (42) **SLE** (22) Other^b^ (22)	–	67.1	Semi-structured interviews	To determine the changes and difficulties experienced by patients due to the side effects of steroids and how they affect their lives
Larsson et al. [26]	2010	Sweden	RA (8) **PA** (3) **SLE** (1) OA (4) Fibromyalgia (2) **Sjogren’s syndrome** (3) Spondyloarthritis (1)	56 (range 35–79)	66.7	Semi-structured interviews	To describe variations in how inpatients with rheumatic diseases perceive drug information provided by a rheumatology nurse
Carpenter et al. [20]	2012	USA	**CSS** (28) **GPA** (132) MPA (18) **Takayasu arteritis** (12) Other^c^ (31)	51 (range 20–82)	91	Semi-structured interviews	To determine issues that negatively impacted patients’ ability to participate in social activities with friends
Applebaum et al. [19]	2013	USA	**SLE** (14) **JIA** (7) Other^d^ (14)	16.9 (range 13–21)	74.3	Focus groups Surveys	To identify the current status of teens embarking on the transition of care process
Cleanthous et al. [21]	2013	UK	**SLE** (17)	44 (range 20–73)	94.1	Structured interviews	To determine aspects of uncertainty in SLE and RA
			RA (15)	57 (range 29–79)	66.7	Semi-structured interviews	
Knight et al. [24]	2016	USA	**SLE** (11) MCTD^f^ (5)	17 (range not given)	81	Semi-structured interviews	To provide a framework for informing subsequent research and care.
Mathias et al. [27]	2017	USA	**SLE** (14)^e^	41.6 (range 19–61)	93	Semi-structured interviews	To develop a comprehensive, SLE-specific, patient-reported outcome measure to assess patient satisfaction with treatment, treatment options, and medical care.

^a^*Abbreviations*: *SLE* Systemic Lupus Erythematosus, *SSc* Systemic Sclerosis, *JIA* Juvenile idiopathic arthriti, *CSS* Churg-Strauss syndrome, *GPA* Granulomatosis with polyangitis, *MPA* Microscopic polyangiitis, *RA* Rheumatoid arthritis, *HSP* Henoch-Schoenlein purpura, *PAN* Polyarteritis nodosa, *PA* Polyarthritis, *OA* Osteoarthritis

^b^Polymyositis, scleroderma, and Sjogren’s syndrome

^c^Undefined

^d^Mixed connective tissue disease, asthma, ulcerative colitis and ten different rheumatologic conditions

^e^Concomittant Vasculitis (2), Lupus nephritis (2), Sjogren’s syndrome (1)

^f^*MCTD* Mixed connective tissue disease

The entries were bolded to show which conditions are SARDs

### Thematic synthesis of SARD patients’ perspectives and experiences with medication use

We thematically synthesized findings across 18 studies identified in our systematic review as well as 22 studies from the aforementioned prior systematic review of patient experiences with SLE [14] that met our inclusion criteria. Altogether thematic synthesis of these 40 studies involved 1522 SARD patients and identified seven predominant, overlapping, and interlocking analytical themes that touch on the perspectives of SARD patients on medication taking: 1) effects of medication on emotional and social well-being, 2) impacts of healthcare provider relationships on treatment, 3) gaining control over treatment, 4) fear and concern with side effects of treatment, 5) understanding the importance of treatment, 6) practical barriers to taking medication, and 7) motivation towards adherence to treatment. We described these themes in detail as follows and outline corresponding descriptive themes and illustrative quotations in Table 2.

**Table 2:** Illustrative Patient Quotations

Analytical Theme	Descriptive Themes	Participant quotes and/or author contribution	Ref
Effects of medications on emotional and social well-being	Emotional aspects of treatment Impacts of medication on life Resentment towards medication Medications serving as reminders of condition Impacts of medications on sexuality Impacts of medications on potential parenthood Emotional impacts of side effects of treatment Embarrassment	“As a wife. Umm, I would say because of it, I am in the process of a divorce. Yeah, many things wouldn’t have happened. I believe so. Physical. Intimate, let’s put it that way. Because like with all the medicines, with all the flares that I got, I mean, I couldn’t be the wife maybe that he wanted me to be. So then things happened. He found somebody else. Umm, he said that that was just because I was not there for him when he needed me ... Because like, when you take so much medicine in your pores, you can smell the medicine coming out of your pores. So, in that way, I know that there was some rejection because of it.”	[23]
		“It was really hard getting to school. I was on prednisone and got pretty fat, so I was getting bullied a lot. It was hard.”	[34]
		“Friends have cancelled dates when they have been ill as they are aware that I need to be careful regarding infections.”	[20]
		“This doctor did not want me to have children in any case. She gave me a lot of medicines and she said “absolutely no children.” [...]But I did not give up. I changed my doctor again, and I found this professor S. She agrees with me, in certain things. She removed some medicines and after some months she said “OK, we can try.” So my daughter arrived.”	[29]
		“I can’t be in the sun… I can’t go to the beach… I would have to wear like a long-sleeved dress… it’s really depressing.”	[24]
Impacts of healthcare provider relationships on treatment	Feeling of being used Feeling of being ignored Desire for continuous care Fear towards treatment Having reliable and/or trustworthy care Desire for information about treatment	“One of the many barriers is that when you feel the doctor is actually not listening to you ... Just, ‘Continue your medication.’ Then I feel pretty upset about it that, it will make me feel I don’t want to continue about doing anything.”	[31]
		“Participants valued highly the ability of doctors to give clear and accurate information about the condition itself, their treatment options and potential side effects of the treatments. Patient 8 noted, ‘... Well, he was just saying if I didn’t take my medicine I was gonna die’.”	[23]
		“I like the information and the explanation of health terms in normal wording. When I get my blood tests back, I don’t know what the terms mean.”	[22]
		“I hate it when they use me as a guinea pig, try other treatments and stuff. They just try me on different immunosuppressant drugs; I really suffer a lot with side effects.”	[34]
Gaining control over treatment	Desire to manage side effects Desire for control over their life Desire for control over the condition	“The prednisone really helped the symptoms, but it made me feel sicker. Like you know the flares went away, but then I was overweight and bloated and you know my joints were swollen from the water. So I felt sicker even though I wasn’t having like you know massive flares.”	[27]
		“Just anything really, to help you to do things for yourself. I mean, I know it is about getting better and all that but there is this sort of isolation aspect and you don’t want to feel that you’ve got something that nobody knows anything about and you can’t find anything about easily.”	[30]
Practical barriers to taking medication	Cost of medications Impact on travel Forgetting to take medications	“It’s one thing to have the illness. That’s bad enough, but the medical bills. .. they’re so much more than I expected and we don’t know how we’re going to pay for all of this.” “Patients with SLE in particular, also reported uncertainty in relation to needing care whilst abroad: I’d be scared that I get sick. I’m just worried about the treatment and healthcare in a different country. [SLE female, 31 years]” “Most patients reported several occasions on which they did not take their medications either because they forgot or because they chose to discontinue, often due to the large quantity of medication they were taking.”	[38]
			[21]
			[42]
Motivation towards adherence to treatment	Medication to continue living Using treatment to improve their prognosis Taking medication out of concern for others	“My hands were black all the time and my feet...And now...look how nice and pink I am.”	[33]
		“Probably the best thing is that it probably helps with more stuff that I can’t actually physically notice, like the stuff that’s stopping my body from fully attacking itself.”	[28]
		“I mean I don’t feel like I’m held back in any way right now. I’m doing everything I want to do – all the clubs and activities I want to do. Psychological health – I would like to think is all there. I mean, sometimes I get stressed out with school and everything, but I feel like everybody does. Yeah, aside from the fact that I’m on medication daily, I consider myself generally – given my circumstances, a generally healthy person, I guess.”	[24]
Understanding the importance of treatment	Belief that their condition isn’t severe enough Lacking understanding about treatment	“It is like so many of them. I have 15 a day that I take. It is annoying. If I think some are less important then I do not take it”	[19]
		“So my problem is that I don’t know if I am bad enough to need it (the medication), I mean you can’t know, so that’s, a struggle.”	[21]
Fear and concern with side effects of treatment	Fear of side effects Non-adherence due to side effects	“But, you know, when it comes to like my energy level I don’t know if that’s the steroids or the Lupus. The memory, I don’t know if that’s really with the two of them, or one ofthem, or…you know, some ofthem I don’t really know if it’s the steroids, the Lupus, or another medication that I might be on.”	[28]
		“[Steroids] just causes more issues with my system. [Steroids] makes it go in chaos. I get more wired up. It’s bad enough I don’t sleep as it is, then I really can’t sleep.”	[28]
		“I haven’t really had any issues with that I know of, but I’m on so many medications I don’t know what a reaction would be with [hydroxychloroquine], so I can’t really say because of all the medications that I’m on.”	[28]
		“Worst thing is the day I received [belimumab] also, you can almost, by the time you leave the doctor’s office you can feel; I’ve talked to other patients there about [belimumab] too, and you can barely make it home; it makes you so tired. I mean you have to drive home after because you have to receive [belimumab] at the doctor’s office…”	[28]

### Theme 1: effects of medications on emotional and social well-being

SARD patients from 24 studies mentioned that medication use impacted emotional and social aspects of their lives, mostly in negative ways [19–21, 23, 24, 26, 28, 29, 31, 32, 34, 37–49]. Patients felt that medications reminded them of their conditions [34]. Adolescent patients with SARDs felt abnormal compared to their peers: *They also felt a strong desire not to take medications in front of their peers, because of the embarrassment of using a pill box and a desire to not appear different from their peers* [19]. The impact of medications on SARD patients’ sexuality and relationships also contributed to negative effects on emotional and social well-being as it was noted that *“because like with all the medicines, with all the flares that I got, I mean, I couldn’t be the wife maybe that he wanted me to be.”* [23]. Related to this, is the impact of medications on potential parenthood as shown in another study of adolescent and adult SLE patients: *“Even if I don’t pass anything on to them, my medications will harm a newborn; it is not something you want to burden your children with.”* [34]. Interviews with patients with SLE revealed how side effects of medications contributed to negative impacts on emotional and social well-being: “*It was really hard getting to school. I was on prednisone and got pretty fat, so I was getting bullied a lot. It was hard*.” [34]. Some vasculitis patients noted that side effects of medications limited their desire to socialize, which resulted in withdrawal from activities, “*I do not want to go out as much or see many people. the predisone has made me moon faced and a weight gain and I feel as if I look like the Cambell Soup kids or Philbury* [sic] *Doughboy”* [20]. Another study of SLE patients found that “the side effects of medication meant the participants had to place boundaries on their future career aspirations” [34], specifically,*“I know that it [lupus] affected what I was doing at university because I wanted to go into archaeology. I went to one of the seminars for it; you have to be in the sun constantly, you have to be 100% healthy, so then I had to change my major.”* [34]. Though most of the noted effects of medications were negative, a study among vasculitis patients identified a positive effect on relationships: *“Now that I am healthy as a result of the right medications, my friendships have gone back to the way they were before.”* [20].

### Theme 2: impacts of healthcare provider relationships on treatment

In 24 studies, SARD patients’ relationships and rapport with their healthcare providers influenced their experiences with medications [19, 21–23, 26, 28–34, 36, 37, 39, 40, 42, 45, 46, 48–52]. Across a number of studies in SLE, patients often felt that they were being used for research when being put on medications, particularly if they were feeling ignored or brushed away by their physicians [28, 31–33, 37, 39, 40, 50, 51], for example, it was noted that *“I hate it when they use me as a guinea pig, try other treatments and stuff”* [34]. Furthermore, when the relationship was not up to patients’ expectations, it often resulted in patients voluntarily discontinuing their medications, *“one of the many barriers is that when you feel the doctor is actually not listening to you… Just, ‘Continue your medication.’ Then I feel pretty upset about it that, it will make me feel I don’t want to continue about doing anything.”* [31]. We also found elements of positive relationships between SARD patients and their rheumatologists. For example, SLE patients appreciate having concise information that they could trust, “…*Well, he was just saying if I didn’t take my medicine I was gonna die*” [23]. Interviews with SLE patients revealed the importance of knowing that their rheumatologist cares and taking the time to explain patients’ conditions and treatments: *“He is trying very hard to help me at least get close to my normal life. For this I am grateful. He explained to me that this type of diagnosis and the medications that go along with it cannot be taken lightly. I wish more doctors could see things that way”* [50]. Also an important finding within this theme is the importance of relationships between SARD patients and allied healthcare providers. For example, SSc patients highly valued their pharmacists’ information regarding their medicines, but also that they were regarded holistically rather than just a medication profile, *“[My pharmacists] are a lot of help to me. I know if I go in and have a question about any of my medicines, you know they’re there for me and they know me, which is another thing.”* [33]. A study among patients with different SARDs highlighted the role of nurses: *They were well cared for and treated in a kindly fashion by the nurse during their hospital stay. It highlighted the importance of someone caring about whether the drug treatment had worked by providing follow up after initial information and treatment had taken place* [26].

### Theme 3: gaining control over treatment

Many studies showed that SARD patients had a strong desire to gain control over their treatment, particularly with respect to the impacts of treatment on three key facets: life, side effects, and their condition [19, 21–23, 27, 30–34, 39, 40, 42, 44, 45, 50, 52–55]. In eight studies, the desire to exert control over SARD patients’ lives was often voiced as a need to a “*normal life.*” Interviews and focus groups with patients with eosinophilic granulomatosis with polyangiitis, granulomatosis with polyangiitis, and polyarteritis nodosa patients revealed that: *Patients appreciate the need for information so they can manage their own drug regime and any side effects* [30].

### Theme 4: practical barriers to taking medication

Practical barriers such as forgetfulness, costs of medications and their restrictive impact on life activities, particularly travel also influenced SARD patients’ opinions towards medications as shown in 12 studies [19, 21, 25, 32, 34, 38, 40, 42, 43, 48, 51, 56]. In a study of adolescents with SLE, authors found forgetting to take medications occurred in many patients “*many teens reported episodes where they had forgotten*” [19]. Cost was another practical barrier as it was noted that *“It’s one thing to have the illness. That’s bad enough, but the medical bills … they’re so much more than I expected and we don’t know how we’re going to pay for all of this.”* [38]. Finally, a study involving focus groups and semi-structured interviews among adolescents and young adults with SLE described the impact of medications on travel: *Young adult participants felt constricted in their ability to travel, because having to rely on medication could leave them stuck somewhere, and because they could not afford the high cost of medication…overseas* [34].

### Theme 5: motivation towards adherence to treatment

In terms of what motivates SARD patients to take their medications as prescribed, we found that using medication to prolong their life and to have their condition improve, particularly out of concern for their families, were important as described in 13 studies [19, 32–34, 37, 39, 40, 42, 44, 45, 51, 54, 55]. Though closely linked to theme 2, *gaining control over treatment*, this theme is distinct in that it represents the reasons for taking medications rather than patients’ desire to feel control over their treatment and not the treatment in control of them. In a study of SLE patients, one patient states how they were adherent to not be selfish after all the time and effort their friends, family and care workers invested in helping them manage their condition: *“people around me, like my mum will take off so much time from work and she will give so much of herself and my dad and just, even like my family friends who I had never met before would give so much of themselves, so I just thought ‘you are being selfish’ (by not taking the tablets), so it kind of clicked, so I just take the drugs.”* [40]. In a study of SSc patients, it was often remarked how the effectiveness of their treatments on their physical appearance could motivate them to be more adherent to their treatments as stated by a patient, *“My hands were black all the time and my feet…And now…look how nice and pink I am.”* [33].

### Theme 6: understanding the importance of treatment

Thirteen studies found that SARD patients’ understanding of their treatments has a profound effect on their perceptions of treatment [19, 21, 23, 34, 39, 40, 42, 44, 46, 51, 52, 55, 57]. This includes the beliefs that their conditions are not severe enough to warrant medication or a lack understanding about their treatments. This often resulted in patients stating that they wondered whether they truly needed to take the medication they were prescribed resulting often in non-adherence and questioning the necessity of medication for them to control their conditions. Part of this had to do with overwhelming amounts of treatment, as noted by a patient who selectively chose which medications were important due to being frustrated at the magnitude of their treatment, *“It is like so many of them. I have 15 a day that I take. It is annoying. If I think some are less important then I do not take it”* [19].

### Theme 7: fear and concern with side effects of treatment

As expected, the impacts of the side effects of treatment were explicitly mentioned by SARD patients in 22 studies [21, 23, 25, 27, 28, 32, 34, 35, 37–49, 51]. This theme mainly recognized perceptions and experiences - notably fear or concern - with side effects of treatment and subsequent influence on non-adherence. This is contrast with theme 2, which touched on the desire to have control over their treatment and associated side effects. An interview-based study among SLE patients noted that this fear of side effects had a significant impact: *Furthermore, some patients were uncertain in relation to the possibility of experiencing serious side effects as a result of their treatment regime, a theme that was often expressed with a sense of concern and worry* [21]. In another study, SLE patients described experiences with side effects as reasons for not taking their medications, *“….whenever I take it, I tend not to feel good, so like every time I keep on buying it, then start like taking two days, I notice I’m getting headache, upset stomach, so I keep some in the fridge, I don’t bother with it, ‘cause it makes me feel worse.”* [51].

## Discussion

We systematically reviewed 18 qualitative studies and thematically synthesized findings across 40 studies involving 1522 SARD patients. With seven analytical themes including 1) effects of medication on emotional and social well-being, 2) impacts of healthcare provider relationships on treatment, 3) gaining control over treatment, 4) fear and concern with side effects of treatment, 5) understanding the importance of treatment, 6) practical barriers to taking medication, and 7) motivation towards adherence to treatment, our thematic synthesis captures the complexities of taking medications for SARD patients. Relevant implications arising from predominant themes include addressing the emotional and social impacts of medications on SARD patients and informing how healthcare providers interact and communicate medication information with SARD patients.

Our thematic synthesis shows that medications largely had negative effects on SARD patients’ and emotional and social well-being. Notably, patients often commented on how they believed medication would affect their hopes of parenthood or their sexual lives. Side effects of medications also contributed to some of these negative impacts on the emotional and social well-being of SARD patients – for example, being bullied at school [34] or not wanting to socialize and withdrawing from activities because of weight gain [20]. As emotional and social impacts of taking medications influence decisions to remain on therapy, addressing them may represent ways to support adherence. For example, emotional impacts may be addressed with peer-based interventions, which have been shown to be effective in other chronic diseases such as schizophrenia, asthma, diabetes, and cystic fibrosis [58–60].

We found that SARDs patients often remarked that their relationship with their rheumatologists explicitly affected their perceptions and experiences with medication taking [31, 50], and SARD patients also valued relationships with allied healthcare providers including nurses and pharmacists. When a good relationship was present, SARDs patients appreciated the ability for providers to effectively relay information such as various treatments and tests, or for providers to succinctly and concisely explain the importance of medication adherence. Our findings also suggest that SARDs patients valued having someone follow them up to see whether their treatment was working as intended [26]. However, patients also had negative experiences with their healthcare providers, including feeling like they were treated as a *“guinea pig”* for research or that their provider did not truly listen to their concerns about their treatment [34]. As a result of a poor relationship, patients lose the motivation to continue treatment, and they lose confidence in those tasked with helping them regain control of their health. Our findings reiterate the importance of healthcare providers establishing good rapport and ensuring clear communication with SARD patients regarding their diagnosis and treatment. Furthermore, with our synthesis also showing that relationships with allied healthcare providers including nurses and pharmacists are as important as relationships with rheumatologists, there are implications for informing collaborative models of care or emphasizing the roles of allied healthcare models in supporting medication taking among patients with SARDs. This maybe particularly relevant given recently demonstrated constraints in access to and availability of rheumatologists [61].

Our study both updates Sutanto et al.’s [14] thematic synthesis of patients’ experiences of living with SLE, though we focused on the experiences of medication taking for a condition rather than experiences with the condition itself. We also expanded on this prior study as it focused on patients with SLE while we considered all patients with SARDs. While there are differences across these conditions, they also share many similarities, including treatments, grouping them as we have done in our study, is important as many of these conditions have been less represented in prior research on medication taking. Our identification of themes on the effects of medication on emotional and social well-being as well as practical barriers such as access to medication when travelling or that forgetfulness influenced medication taking, represent novel contributions that expand on this prior work.

Strengths and limitations of our study deserve comment. We overcame the inherent limitation of qualitative research studies, that is, small sample sizes by combining them using the technique of thematic synthesis, thus resulting in qualitative research on SARD patients’ perspectives on medication taking using an effectively much larger sample size. However, while our search strategy did attempt to capture studies with SARDs patients, the paucity of medication use studies in certain SARDs resulted in a limitation of our study. Also a potential limitation is that we did not conduct quality appraisal of included studies. However, there is considerable debate on the relevance of quality appraisals when the goal of qualitative synthesis is to address aforementioned limitations of quality research studies [62]. Some included studies also lacked transparency in describing their patient samples resulting in our inability to distinguish which SARD a patient was diagnosed with, as well other concurrent conditions the patient might have. As with any other systematic review, the inclusion of relevant studies may have been limited by publication bias. However, unlike quantitative meta-analyses, the sample of a qualitative synthesis is purposive rather than exhaustive, as the aim is to provide interpretive explanation rather than prediction [12, 63]. Finally, an inherent limitation of thematic syntheses is that the line-by-line coding, identification of descriptive themes, and generation of analytical themes is a subjective process. Nevertheless, we attempted to minimize this limitation by having each step performed independently by separate reviewers.

## Conclusions

In conclusion, our systematic review and thematic synthesis highlights the complexities of taking medications among patients with SARDs. Given the paucity of existing adherence interventions targeting this patient population, our review has certain practical implications for care, namely the need to address emotional and social impacts of medication use and the necessity of establishing a meaningful and trusting professional relationship with patients.

## Additional file

Additional file 1: Search strategies; A list of search terms used for each database searched. (DOCX 19 kb)

## Abbreviations

CSS: Churg-Strauss syndrome
EGPA: Eosinophilic granulomatosis with polyangiitis
GPA: Granulomatosis with polyangitis
HSP: Henoch-Schoenlein purpura
JIA: Juvenile idiopathic arthritis
MCTD: Mixed connective tissue disease
MPA: Microscopic polyangiitis
OA: Osteoarthritis
PA: Polyarthritis
PAN: Polyarteritis nodosa
RA: Rheumatoid arthritis
SARD: Systemic autoimmune rheumatic disease
SLE: Systemic lupus erythematosus
SSC: Systemic sclerosis
