# Supplementary material for: How do patients with systemic autoimmune rheumatic disease perceive the use of their medications: a systematic review and thematic synthesis of qualitative research
Source: BMC Rheumatol. 2018 Apr 2;2:9. doi: 10.1186/s41927-018-0017-8 (PMC6390776; doi:10.1186/s41927-018-0017-8)
Supplement: Supplementary file 1 — Search strategies; A list of search terms used for each database searched. (DOCX 19 kb) [file 41927_2018_17_MOESM1_ESM.docx]

**SEARCH STRATEGIES**

**Medline Search Strategy**

1. Lupus Erythematosus, Systemic/ or Lupus Erythematosus, Systemic.mp.
2. Lupus Nephritis/ or Lupus Nephritis.mp.
3. Lupus Vasculitis, Central Nervous System/ or Lupus Vasculitis, Central Nervous System.mp.
4. Lupus.mp.
5. Myositis/ or Myositis.mp.
6. Dermatomyositis/ or Dermatomyositis.mp.
7. Polymyositis/ or Polymyositis.mp.
8. Wegener*.mp.
9. Granulomatosis with Polyangiitis/ or Granulomatosis with Polyangiitis.mp.
10. Giant Cell Arteritis/ or Giant Cell Arteritis.mp.
11. Takayasu arteritis/ or Takayasu arteritis.mp.
12. Polyarteritis Nodosa/ or Polyarteritis Nodosa.mp.
13. Or/1-12
14. Qualitative Research/
15. Nursing Methodology Research/
16. Questionnaires/
17. exp Attitude/
18. Focus Groups/
19. discourse analysis.mp.
20. content analysis.mp.
21. ethnographic research.mp.
22. ethnological research.mp.
23. ethnonursing research.mp
24. constant comparative method.mp.
25. qualitative validity.mp.
26. purposive sample.mp.
27. observational method$.mp.
28. field stud$.mp.
29. theoretical sampl$.mp.
30. phenomenological research.mp.
31. life experience$.mp.
32. cluster sampl$.mp.
33. or/14-32
34. 13 and 33
35. Limit 34 to English language

**Embase Search Strategy**

1. Lupus Erythematosus, Systemic/ or Lupus Erythematosus, Systemic.mp.
2. Lupus Nephritis/ or Lupus Nephritis.mp.
3. Lupus Vasculitis, Central Nervous System/ or Lupus Vasculitis, Central Nervous System.mp.
4. Lupus.mp.
5. Myositis/ or Myositis.mp.
6. Dermatomyositis/ or Dermatomyositis.mp.
7. Polymyositis/ or Polymyositis.mp.
8. Wegener*.mp.
9. Granulomatosis with Polyangiitis/ or Granulomatosis with Polyangiitis.mp.
10. Giant Cell Arteritis/ or Giant Cell Arteritis.mp.
11. Takayasu arteritis/ or Takayasu arteritis.mp.
12. Polyarteritis Nodosa/ or Polyarteritis Nodosa.mp.
13. Or/1-12
14. Qualitative Research/
15. Nursing Methodology Research/
16. Questionnaires/
17. exp Attitude/
18. Information processing/
19. discourse analysis.mp.
20. content analysis.mp.
21. ethnographic research.mp.
22. ethnology.mp.
23. ethnonursing research.mp
24. constant comparative method.mp.
25. qualitative validity.mp.
26. purposive sample.mp.
27. observational method$.mp.
28. field stud$.mp.
29. theoretical sampl$.mp.
30. phenomenology/
31. phenomenological research.mp.
32. life experience$.mp.
33. cluster sampl$.mp.
34. or/14-33
35. 13 and 34
36. Limit 35 to English language

**Social Sciences Citation Index (SSCI) Search Strategy**

1. Systemic Lupus Erythematosus
2. Lupus Nephritis
3. Lupus Vasculitis
4. Myositis
5. Dermatomyositis
6. Polymyositis
7. Granulomatosis with Polyangiitis
8. Wegener*
9. Giant Cell Arteritis
10. Takayasu Arteritis
11. Polyarteritis Nodosa
12. Or/1-11
13. Qualitative Research
14. Nursing Methodology Research
15. Questionnaires
16. Attitude
17. Focus Groups
18. Discourse Analysis
19. Content Analysis
20. Ethnographic Research
21. Ethnological Research
22. Ethnonursing Research
23. Constant Comparative Method
24. Qualitative Validity
25. Purposive Sample
26. Observational Method*
27. Field Stud*
28. Theoretical Sampl*
29. Phenomenology
30. Phenomenological Research
31. Life Experienc*
32. Cluster Sampl*
33. Or/13-32
34. 12 and 33
35. Limit to English

**Cumulative Index to Nursing and Allied Health Literature (CINAHL) Search Strategy**

1. Lupus Erythematosus, Systemic OR MH Lupus Erythematosus, Systemic
2. Lupus Nephritis OR MH Lupus Nephritis
3. Lupus Vasculitis, Central Nervous System OR MH Lupus Vasculitis, Central Nervous System
4. Lupus OR MH Lupus
5. Myositis OR MH Myositis
6. Dermatomyositis OR MH Dermatomyositis
7. Polymyositis OR MH Polymyositis
8. Wegener OR MH Wegener
9. Granulomatosis with Polyangiitis OR MH Granulomatosis with Polyangiitis
10. Giant Cell Arteritis OR MH Giant Cell Arteritis
11. Takayasu Arteritis OR MH Takayasu Arteritis
12. Polyarteritis Nodosa OR MH Polyarteritis Nodosa
13. Qualitative Research
14. Nursing Methodology Research
15. Questionnaires
16. Attitude
17. Focus Groups
18. Discourse Analysis
19. Content Analysis
20. Ethnographic Research
21. Ethnological Research
22. Ethnonursing Research
23. Constant Comparative Method
24. Qualitative Validity
25. Purposive Sample
26. Observational Method*
27. Field Stud*
28. Theoretical Sampl*
29. Phenomenology
30. Phenomenological Research
31. Life Experienc*
32. Cluster Sampl*
33. Or/S1-S12
34. Or/S13-S32
35. S33 and S34 – Narrow by Language: -english
